# Saline Injections in Diptheritis

**Published:** 1860-01

**Authors:** 


*Saline Injections in Diptheritis.*

M. Roche states that he has been successful in some cases in which he has tried the injection of a solution of chloride of sodium into the throat, that in his next case he is disposed to employ it as the sole means of treatment. He practices, in fact, a continuous, or almost continuous, irrigation of the throat, by means of Eguisier's irrigator, provided with a canula having a very small jet. He believes that it is in such irrigations, whether employing salt, alum, or the chlorates, we should seek for curative agents.—*Union Medicale.*

